# Severe obstructive sleep apnea treatment with mandibular advancement device: A case report

**DOI:** 10.5935/1984-0063.20180022

**Published:** 2018

**Authors:** Maria de Lourdes Rabelo Guimarães, Ana Paula Hermont, Thais Moura Guimarães, Cibele Dal-Fabbro, Lia Bittencourt, Cauby Maia Chaves Junior

**Affiliations:** 1 Universidade Federal de Minas Gerais, Faculdade de Odontologia - Belo Horizonte, MG, Brasil.; 2 Universidade Federal de São Paulo, Departamento de Psicobiologia - São Paulo, SP, Brasil.; 3 Universidade Federal do Ceará, Departamento de Clínica odontológica - Fortaleza, CE, Brasil.

**Keywords:** Sleep Apnea, Obstructive, Polysomnography, Mandibular Advancement

## Abstract

Mandibular advancement device (MAD) has been described as an alternative treatment to the severe obstructive sleep apnea (OSA), once it is not as effective as the continuous positive airway pressure therapy (CPAP) in reducing the apnea and hypopnea index (AHI). The objective of this study is to report a case using a MAD in a CPAP-intolerant patient suffering from severe OSA. Polysomnography exams were performed before and after treatment. Five months after fitting and titrating the MAD, the AHI was reduced from 80.5 events/hour to 14.6 events/hour and the minimum oxyhemoglobin saturation (SpO_2_) increased from 46% to 83%. A two-year assessment of therapy revealed an AHI of 8 events/hour and SpO_2_ of 85%.

## INTRODUCTION

Obstructive sleep apnea (OSA) is a respiratory disorder characterized by recurrent episodes of total or partial obstruction of the upper airway during sleep^1^. Intermittent hypoxemia, transient hypercapnia, and frequent arousals are also consequences of this disorder^1^. The signs and symptoms of OSA are commonly described as excessive sleepiness, cognitive impairment, cardiovascular disease, mood changes and metabolic dysfunction^2^.

Severe OSA has been associated with a greatest risk factor for atherosclerosis, acute myocardial infarction and general mortality, when compared to mild and moderate OSA^3^^-^^5^. There is also a positive relationship between apnea/hypopnea index (AHI) and the presence of these outcomes^3^^-^^5^. Therefore, the treatment of severe OSA is very important, even if the complete resolution of AHI was not achieved^5^.

The continuous positive airway pressure (CPAP) therapy is the most efficient treatment for OSA and improves patient’s subjective symptoms and cardiometabolic alterations^3^^,^^6^. Population studies have observed that CPAP treatment is related to decreased cardiometabolic risk in subjects with severe OSA^3^. Nevertheless, the CPAP adherence is an important limitation of the treatment. Approximately 46 to 83% of patients do not use CPAP for more than 4 hours a day, which leads to the need of an alternative treatment^7^.

The mandibular advancement device (MAD) is considered an alternative treatment for CPAP^8^. Despite the greater patient compliance to the therapy (76 to 86%)^9^^,^^10^ it is not as effective as CPAP in improving the AHI^11^. Studies have detected that 37% up to 42.6% of patients on MAD therapy achieves a success response rate (the reduction of AHI <5 events/hour)^9^^,^^12^^,^^13^. In severe OSA, success with MAD is lower, only 22% to 23% of patients have complete resolution in AHI^12^^,^^13^. However, a study referring mortality in severe OSA population found that CPAP-intolerant individuals treated with MAD died less than non-treat patients^5^. These observations reinforce the importance of treating severe OSA patients even without complete resolution of AHI^5^. The purpose of this study is to report a successful case using a mandibular advancement device (MAD) in a CPAP-intolerant individual with severe OSA.

## CASE REPORT

### Patient data

A 49-year-old CPAP-intolerant male patient was referred by an otorhinolaryngologist for MAD treatment. In the anamnesis, no orthodontic, orthopedic or surgical intervention was reported in the craniocervical complex. The patient’s main complaint was excessive daytime sleepiness, persistent fatigue, frequent and loud snoring and witnessed apneas. He scored 10 points in the Epworth Sleepiness Scale^14^ and presented a body mass index (BMI) of 32.9 kg/m^2^. In basal PSG, the patient presented a sleep efficiency of 80.6%, AHI of 80.5 events/h (apnea index = 36.1, hypopnea index = 44.4). The mean of SpO_2_ was 93%, the minimum of SpO_2_ was 46%, and the percentage of time below 90% was 32.7%. Regarding the sleep architecture, it presented 4.3% of N3, 7.4% of REM and 64.3/h of arousal index.

### Polysomnography

The patient did two full night polysomnography recordings: the baseline recording, and with MAD titrated in situ. A type III home sleep portable monitor, the ApneaLink, was also used to monitor the patient. This device records 4 channels from 3 non-invasive sensors which measure respiratory effort, airflow, pulse rate, and oxygen saturation.

The full night polysomnography (PSG) was performed in a sleep laboratory. Polysomnography included electroencephalography, electromyography, electrocardiogram, oxygen saturation measured by a finger pulse oximeter and electroculogram. The respiratory variables recorded by pressure nasal cannula and thermistor. Respiratory effort was measured using a respiratory inductance plethysmography. Snoring was recorded by a microphone and body position was monitored using a piezoelectric sensor.

Polysomnographic recordings were scored according to the guidelines of the American Academy of Sleep Medicine^1^. Obstructive apnea was defined as a = 10-second cessation of air flow on the pressure nasal cannula, associated with an oronasal thermal sensor. Hypopnea was defined as a = 50% reduction in airflow, or a reduction of airflow <50% on the nasal pressure cannula accompanied by a decrease = 3% in oxygen saturation (SpO_2_) or an arousal. Central apnea was defined by the absence of respiratory effort throughout the entire period of absent air flow; and mixed apnea was defined by the onset of the respiratory event with no airflow and no respiratory effort during the first half of the event and, at the second half of the event, the absence of airflow persisted even after a resumption of inspiratory effort. The minimum SpO_2_ (SpO_2_ nadir) was also recorded^1^.

### Mandibular advancement device

Complete orthodontic documentation was requested, including cephalometric analysis (Table 1). The patient presented satisfactory dental and periodontal conditions and was capable to perform protrusive, latero-protrusive, opening and closing mandibular movements in a coordinated way. The oropharyngeal inspection revealed an elongated soft palate, Mallampati Grade IV and palatine tonsil Grade II.

**Table 1:** Cephalometric analysis.

Cephalometric measures	Obtained value	Normal values (mean + SD)
Anterior cranial base	77.55 mm	80.00±2.00 mm
Maxilla length (ENA-ENP)	49.00 mm	55.80±3.00 mm
Mandible length	123.00 mm	125.77 mm
Mandibular ramus length	65.83 mm	53.00 mm
Mandibular body length (Go-Pog)	87.68 mm	82.80±3.60 mm
S-N.A	76.53 º	81.50 º±3.20 º
S-N.B	78.00 º	79.40 º±2.90 º
A-N.B	-1.47 º	2.10 º±1.90 º
S-N.GN	64.44 º	66.00 º±3.20 º
N-A.Pog	-6.53 º	2.80 º±2.20 º
Superior pharyngeal airway space	7.24 mm	11.50±2.00 mm
Lower pharyngeal airway space	6.55 mm	11.00 ±2.00 mm
Soft palate length	46.04 mm	37.00±3.00 mm
Distance of hyoid bone- third vertebrae	49.20 mm	40.00±5.00 mm

**Figure 1:**
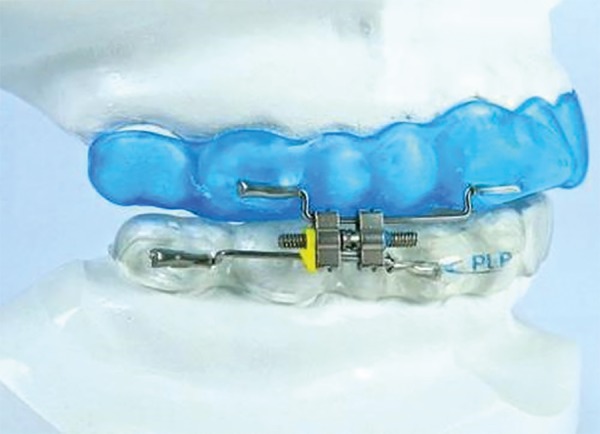
Mandibular advancement device.

The treatment was conducted with a MAD (Lateroprotrusive Plate - PLP^®^) (Figure 1). The absolute range of maximal mandibular protrusion was measured (in mm) with the use of the George Gauge (Great Lakes Orthodontics, Ltd., New York, USA). The construction bite was registered at 50% of the maximum mandibular protruded position (patient’s maximum protrusion was 9.0 mm) and progressive advances were performed up to 7mm. In this position the MAD was optimally titrated resulting in symptoms resolution and the patient reported no complaints of symptoms in the temporo-mandibular joints.

The time interval between fitting the MAD and the monitoring PSG exam with the MAD was 5 months. After fitting and titrating the MAD the patient continued to return to the annual follow-up visits. There were no complaints related to temporomandibular disorders or masticatory muscles. However, there were discrete alterations in dental occlusion, which were carefully managed.

## RESULTS

The results of the full night polysomnography are shown in Table 2. There was improvement in AHI, from 80.5 events/h to 14.6 events/h, the SpO_2_ nadir increased from 46% to 83% and the SpO_2_ <90% decreased from 32.7% to 1.06%.

**Table 2:** Baseline and after treatment (control) polysomnographic parameters.

					SpO_2_					
Parameters	AHI	AI	HI	X	Min	% time<90%	Sleep efficiency	N3 (%)	REM (%)	Arousal/h
Basal PSG	80.5	36.1	44.4	93%	46%	32.78	80.6	4.3	7.1	64.3
Control PSG	14.6	0.9	13.6	95%	83%	1.06	87.9	6.8	7.4	15.8
2-year follow up (ApneaLink)	8.0	3.0	5.0	96%	85%	1.00	-	-	-	-

PSG=polysomnography. AHI=Apnea and Hypopnea Index. AI=Apnea Index. HI=Hipopneia Index. X=mean oxygen saturation. Min=Minimum saturation. % Time <90%=Percentage of time that saturation remained below 90%. OA=Oral appliance.

After fitting and titrating the MAD the patient continued to return to the annual follow-up visits. The patient reported improvement in sleep quality and in his quality of life, presenting more disposition for his daily activities, without daytime sleepiness (the patient scored 8 points in the Epworth Sleepiness Scale after the treatment, the baseline score was 10 points) and with occasional snoring. After 2 years of follow-up, the patient refused to do the control polysomnography, therefore, the control was performed with the ApneaLink Plus Home Sleep Screening Device (ResMed). The results showed an AHI of 8 events/h and minimum SpO_2_ of: 85%. The compliance of MAD was 7h per night. It was measured subjectively through patient reporting at follow-up visits.

During the follow-up, it was necessary to replace the OA once and repair it twice due to fracture of one of the plates. During the first year of treatment the patient presented mild pain in the masticatory musculature and after 4 years using the MAD the patient’s overjet and overbite decreased by 1.5 mm. There was no need to discontinue the MAD use or to do orthodontic interventions.

## DISCUSSION

The patient presented in the basal PSG an AHI of 80.5 events/h and SpO_2_ nadir of 46%. Before initiating the therapy with the MAD, the patient tried CPAP for a few nights, once it is the primary treatment indication for severe sleep apnea. Nevertheless, he abandoned its use; discomfort was the reason for noncompliance.

After titrating the MAD, the results of the control polysomnographic, with the oral appliance in situ showed an objective decrease in the rates of respiratory obstructive events. The full night PSG showed an AHI = 14.6 events/h and SpO_2_ = 83%. The control conducted with the ApneaLink portable monitoring system after two years of treatment showed an AHI = 8 events/h and an improvement in the SpO_2_ nadir = 85%. There was also an improvement in the proportion of time with SpO_2_ <90%, that decreased from 32.87% to 1.06%, and the arousal index decreased from 64.3 events/h to 15.8 events/h.

Patients with severe OSA have an increased cardiovascular risk. Without CPAP adherence, they must be treated with alternative therapies even if they remain with some degree of residual AHI^5^. Some studies suggest that the greater adherence to MAD therapy may compensate the non-complete resolution of the apnea and hypopnea events^5^. A systematic review showed that mild OSA may have a minimal impact in the patient’s general health^15^. Therefore, a possible residual AHI related to the MAD therapy (studies presents a mean post-treatment residual AHI of 5 to 15 events/h) maybe does not have significant impacts on the patient’s general health^5^^,^^16^^,^^17^.

Despite the significant improvement in AHI and micro-arousal, the patient described in this study did not show significant improvement in the sleep architecture as observed by the N3 and REM stages, which increased from 4.3% to 6.8% and 7.1% to 7.4%, respectively. This result is consistent with the latest review by the American Academy of Sleep Medicine and the American Academy of Dental Sleep Medicine that found no significant changes in sleep architecture with MAD therapy^11^.

The cephalometric analysis showed some characteristics that could jeopardize success outcomes with MAD such as a decrease in maxillary length (ENA-ENP) and a maxillary retroposition (ANS). The described patient also presented other features that were not favorable to the intraoral appliance treatment such as an elongated uvula, increased soft palate length and a lower displaced hyoid bone which contributed to the narrowing of the airway and the possibility of pharyngeal obstruction during sleep. On the contrary, the measurements of the lower airspace of the pharynx were diminished, which according to a study carried out by Cunha *et al.*^18^ is a good predictor of success for MAD therapy.

The main objective of MAD therapy is to reduce or normalize the AHI. There are some predictors that help dentists in the attempt to identify which patients will benefit more from the treatment. However, the predictors are still not fully clinically reliable. In the present case the patient was 49 years old, obese, presented lower mandibular advancement (7mm) and higher AHI. This outcome was not compatible with some strong predictors such as lower AHI, lower age, lower BMI and higher mandible protrusion^12^^,^^13^. This data corroborates the questioning of factors predicting the most suitable individuals for the treatment with MAD and stimulates sleep professionals to conduct the treatment even if the patient presents negative predictors.

Attention should be given to follow-up visits and PSG or home-based monitoring exams for sleep apnea detection^11^. These tests can provide objective measures related to the long-term effectiveness of the OSA treatment. Although the patient in this report presented excellent compliance (7h/day during 7 days/week) to the MAD treatment and performed regular follow-up visits, the difficulty consisted in convincing him to perform the control PSG exams. The solution found was the use of home tests (ApneaLink) to minimize the risk of under-treatment. It is also worth mentioning that BMI and information about supine and non-supine AHI were not recorded in both post-treatment control evaluations. As mentioned before, these are important data that might be related to predictors of treatment success.

The case reported in this article showed an improvement in the patient’s health, who initially presented a severe OSA (AHI: 80.5 events/h) and after the successful MAD therapy, the AHI decreased to 14.6 events/h), demonstrating a good response rate.

It is worth noticing that a multidisciplinary approach including phonoaudiology treatment can tonify pharyngeal musculature and reduce the possibility of pain complaints related to the masticatory muscles during the mandibular advancement. Furthermore, behavioral changes such as weight loss could corroborate to a decrease in the patients’ AHI.

## CONCLUSION

The mandibular advancement device improved the polysomnographic parameters in a case of severe OSA and these effects were maintained during the 2-year follow-up.
